# Supplementary material for: PoPI: A machine learning-based consensus mechanism for blockchain-enabled IoT systems
Source: PLoS One. 2026 Apr 13;21(4):e0347124. doi: 10.1371/journal.pone.0347124 (PMC13075720; doi:10.1371/journal.pone.0347124)
Supplement: Attachment — Submitted filename: _Response_to_Reviewers__PoPI__Plos_One_.pdf [file pone.0347124.s001.pdf]

Original Manuscript ID: PONE-D-26-01815

Original Manuscript Title: "PoPI: A Machine Learning-Based Consensus Mechanism For Blockchain-Enabled IoT Systems"

To: Dr. YANG (JACK) LU

Re: Response to reviewers

Dear Editor,

Thank you for allowing a resubmission of our manuscript, with an opportunity to address the reviewers' comments.

We are uploading (a) our point-by-point response to the comments (below) (Response to Reviewers), (b) an updated manuscript with yellow highlighting indicating changes (*Revised Manuscript with Track Changes*), and (c) a clean updated manuscript without highlights (*Manuscript*).

Best regards,

Hossen et al.

Reviewer#2, Concern #1: There should be a literature review in Related Works.

Author response: We thank the reviewer for this insightful suggestion. We agree that a clearer literature review is essential to properly position the proposed work within the existing body of research. We also acknowledge that some parts of the current Related Works section did not sufficiently describe the working principles of certain consensus mechanisms. To address this, we have revised and expanded the section to provide a more structured discussion of both traditional and modern blockchain consensus approaches, and we have added additional analysis highlighting their limitations in IoT environments.

Author action: The Related Works section (Page 6-7) has been revised to strengthen the literature review and explicitly highlight the research gap that motivates the proposed consensus mechanism.

Reviewer#2, Concern #2: Although the authors have added ablation studies, the result analysis remains at the level of performance indicators and lacks an in-depth explanation of the internal working principles of the mechanism.

Author response: We thank the reviewer for this valuable comment. We agree that the results of the ablation study could benefit from a more detailed discussion of the underlying mechanisms responsible for the observed outcomes. Additionally, we have carefully evaluated and revised the text in the Result Analysis section so that for each reported performance level, a corresponding discussion is provided to explain the working principles behind the observed results.

In addition, to help better understand the working principles of PoPI and related mechanisms, we have added a new subsection titled *Comparative Analysis* in the *Theoretical Analysis* section. This subsection includes a new figure (Fig 4) illustrating how successive consensus approaches have progressively added capabilities to block producer selection, and a new table (Table 2) providing a detailed comparison of PoPI with existing ML-based consensus mechanisms. While this discussion remains primarily at a theoretical level, it offers additional context that explains the observed results more comprehensively.

Author action: The *Ablation Study* in *Result Analysis* (Page 38-39) section has been revised for in-depth explanation, and a new *Comparative Analysis* (Page 27-28) subsection with figure (Fig 4, Page 28) and table (Table 2, Page 28) has been added to provide further insight into the mechanism's working principles compared to other mechanisms.

Reviewer#2, Concern #3: There should be a discussion of the limitations of the manuscript in the Conclusion.

Author response: We thank the reviewer for this valuable suggestion. We agree that explicitly discussing the limitations of the proposed work strengthens the manuscript. To address this, we have added a dedicated discussion in the Conclusion section that highlights key limitations, including the use of a custom simulation environment, the reliance on federated learning for model maintenance and training, and the suitability of the chosen machine learning paradigm for evaluating node reliability. These additions clarify the scope and constraints of the current work while guiding directions for future improvements.

Author action: The Conclusion section (Page 43) has been revised to include a discussion of the limitations of the manuscript and to outline the corresponding future research directions.

Reviewer#4, Concern #1: The paper proposes modeling block producer selection as a supervised learning regression problem and provides a basic mathematical form and loss function framework. However, the theoretical justification for model selection, feature importance, and training data sources is insufficient. It lacks a clear explanation of the theoretical basis or performance comparison for the final model selection, and also lacks discussion on feature contribution or model interpretability, which is particularly important in security-sensitive scenarios like blockchain consensus mechanisms. It is recommended to add an explanation of the rationale for selecting the machine learning model, supplement it with feature importance or interpretability analysis, and provide a more rigorous theoretical explanation of the source and reliability of the model training data.

Author response: We thank the reviewer for this insightful and constructive comment. We agree that further clarification of the rationale behind model selection, feature design, and training data is important, particularly in the context of security-sensitive blockchain consensus mechanisms.

For evaluating PoPI within the simulation framework, we adopt a Linear Regression (LR) model. This choice is motivated by its low computational overhead and strong scalability with increasing numbers of nodes, which are essential properties for resource-constrained IoT environments.We have clarified this rationale in the *Consensus Model Structure* subsection within the main section describing the proposed consensus mechanism. In addition, we provide experimental support in the *Model Resource Consumption* section, where results show that LR maintains stable model size and consistent inference time as the number of nodes increases. We note that, in practice, the choice of the underlying model can be adapted based on application-specific requirements, as different use cases may impose varying trade-offs between reliability and efficiency.

Regarding feature design, we agree that providing clearer guidance is beneficial. While feature importance may vary across deployments and is therefore not fixed within the framework, the proposed approach is designed to flexibly accommodate a wide range of reliability and performance-related features. To better support this, we have added a new table (Table 1) that categorizes potential features into performance history, hardware and network availability, configuration reliability, participation fairness, and block-specific information. Each category reflects a distinct aspect of node behavior, and representative features are provided to guide practical implementation. A corresponding discussion has been included in a new subsection titled Feature Selection.

To further clarify the source and reliability of the training data, we have expanded the *Continuous Model Training* section. In each block production cycle, nodes either successfully generate a block or fail, and this outcome serves as the label, while the features used during inference form the inputs for retraining. Since this information is recorded on-chain by supervisor nodes, as defined in the PoPI design, it is transparent, tamper-resistant, and can be independently verified by all participants. This significantly reduces the risk of manipulated training data and improves the reliability of the learning process.

Author action: The manuscript has been revised to explicitly justify the selection of the Linear Regression model in the *Consensus Model Structure* subsection (Page 18), introduce a new table (Table 1, Page 20) and a dedicated *Feature Selection* subsection (Page 19-20) to provide structured guidance on feature design, and expand the *Continuous Model Training* section (Page 21) to clarify the source, construction, and reliability of the training data.

Reviewer#4, Concern #2: The security analysis section of the paper mainly explains the advantages of PoPI, such as unpredictability, incentive mechanisms, and model update mechanisms, from a theoretical perspective. However, the related analysis remains at the conceptual level. For example, the paper lacks systematic analysis or experimental verification of common blockchain attack scenarios. Furthermore, since the consensus mechanism relies on machine learning models for node selection, if attackers influence the model input by forging node state data, it could pose a potential risk to system security; this issue is discussed only briefly in the paper. Recommendation: 1. Add theoretical analysis or simulation experiments for typical attack scenarios to the security analysis section, and further explain PoPI's defense capabilities against these attacks.

Author response: We thank the reviewer for this valuable suggestion. We agree that a more detailed discussion of PoPI's resilience to typical blockchain attacks strengthens the manuscript.

In response, we have added a new subsection titled *Attack Mitigation* in the Security Analysis section. This subsection discusses major attack vectors, including Sybil attacks, collusion attacks, liveness attacks, DDoS attacks, and fork attacks [1]. In this section, we theoretically explain how PoPI mitigates these threats through adaptive, feature-driven node evaluation and dynamic selection mechanisms. Regarding potential manipulation of model inputs, the discussion has been elaborated in the *Incentive Mechanism* subsection, which clarifies how recording ML inputs on-chain enables data verification and encourages participants to maintain honest behavior by accurately reporting node states.

Author action: A new subsection titled *Attack Mitigation* (Page 25-26) has been added to the Security Analysis

section, and the discussion of model input integrity has been expanded in the *Incentive Mechanism* (Page 25) subsection.

Reviewer#4, Concern #3: Although the paper proposes that PoPI predicts node capabilities and periodically selects block producer groups through machine learning, its overall framework still shares some similarities with existing ML-based consensus mechanisms. The current paper's comparison focuses more on performance metrics, while the explanation of the essential innovations at the mechanism level is insufficient. This may make it difficult for readers to clearly understand PoPI's core contributions compared to existing methods. It is recommended to add a clearer comparative analysis table to the relevant work section, highlighting the essential differences between PoPI and existing ML-based consensus algorithms in terms of mechanism design, dynamic feature modeling, and node fairness mechanisms, and further emphasize the theoretical contributions in the discussion section.

Author response: We thank the reviewer for this constructive recommendation. We agree that highlighting PoPI's core contributions compared to existing ML-based consensus mechanisms would strengthen the manuscript and improve clarity for readers.

In response, we have added a new subsection titled *Comparative Analysis*. In this subsection, we first illustrate the evolution of consensus mechanisms in a new figure (Fig 4), showing how successive approaches progressively added new capabilities to block producer selection. Traditional mechanisms such as PoW and PoET provide security and reduced computational demands, respectively, but face limitations in energy efficiency and scalability. Machine learning-based mechanisms, such as PoEM, improve selection intelligence but incur high inference overhead and do not adapt to dynamically changing node states. In contrast, PoPI leverages a periodic selection model and incorporates dynamic node features through supervisors, achieving improved adaptability and scalability in dynamic IoT environments.

Additionally, a new table (Table 2) provides a detailed comparison of PoPI with existing ML-based consensus mechanisms, including PoDL, TDCB-D3P, CE-PBFT, and PoEM. Column-wise, the table highlights the type of model used, its purpose, usage pattern, whether dynamic features are incorporated, and the fairness of node selection. This comparison clearly shows that PoPI is the only mechanism that periodically applies the model, integrates dynamic node features, and introduces fairness-aware criteria, going beyond mere historical performance to ensure equitable participation. Overall, these additions emphasize the theoretical and practical contributions of PoPI in mechanism design, dynamic feature modeling, and fairness, distinguishing it from prior ML-based approaches.

Author action: A subsection *Comparative Analysis* (Page 27-28), along with a new figure (Fig 4, Page 28) and a new table (Table 2, Page 28), has been added to the manuscript to clearly illustrate PoPI's unique contributions in mechanism design.

References

[1] A. Guru, B. K. Mohanta, H. Mohapatra, F. Al-Turjman, C. Altrjman, and A. Yadav, "A survey on consensus protocols and attacks on blockchain technology," *Applied sciences*, vol. 13, no. 4, p. 2604, 2023.
