# Supplementary material for: PoPI: A machine learning-based consensus mechanism for blockchain-enabled IoT systems
Source: PLoS One. 2026 Apr 13;21(4):e0347124. doi: 10.1371/journal.pone.0347124 (PMC13075720; doi:10.1371/journal.pone.0347124)
Supplement: Attachment — Submitted filename: _Response_to_Reviewers__PoPI__Plos_One.pdf [file pone.0347124.s002.pdf]

Original Manuscript ID: PONE-D-26-01815

Original Manuscript Title: “PoPI: A Machine Learning-Based Consensus Mechanism For Blockchain-Enabled IoT Systems”

To: Dr. YANG (JACK) LU

Re: Response to reviewers

Dear Editor,

Thank you for allowing a resubmission of our manuscript, with an opportunity to address the reviewers’ comments.

We are uploading (a) our point-by-point response to the comments (below) (Response to Reviewers), (b) an updated manuscript with yellow highlighting indicating changes (*Revised Manuscript with Track Changes*), and (c) a clean updated manuscript without highlights (*Manuscript*).

Best regards,

Hossen et al.

Reviewer#1, Concern #1: This manuscript proposes PoPI, a machine-learning–based consensus mechanism for blockchain-enabled IoT systems that periodically selects a group of block producers and randomizes block production order within the group. The motivation is clear and relevant, and the overall system architecture is thoughtfully designed. The paper is generally well organized, readable, and technically coherent.

That said, while the approach is promising, there are several issues that currently limit how strongly the experimental results support the authors' broader claims.

Technical soundness and experimental support. The idea of amortizing ML inference across multiple blocks and incorporating dynamic device-level features is reasonable, and the simulation results suggest improvements in latency, throughput, and participation efficiency relative to the chosen baselines. However, all evaluations are conducted using a custom discrete-event simulator with modeling assumptions defined by the authors. There is no validation using real IoT traces, real blockchain implementations, or alternative simulation frameworks. As a result, it is difficult to assess how robust the reported gains would be under different or less idealized conditions. Claims regarding scalability and real-world applicability would therefore benefit from more cautious framing or additional supporting evidence.

Author response: We thank the reviewer for this insightful comment.

In this study, we adopted a custom discrete-event simulator to ensure controlled experimentation, reproducibility, and systematic evaluation under varying network and device-level conditions. The simulator allows us to isolate the impact of the proposed amortized ML inference and dynamic device-level feature integration without interference from implementation-specific artifacts. We have reported detailed modeling assumptions and parameter settings to enhance transparency and reproducibility.

However, we acknowledge that simulation-based evaluation cannot fully capture all real-world deployment complexities. Although our results demonstrate improvements under diverse simulated conditions, we agree that validation using real IoT traces and production-grade blockchain platforms would provide stronger evidence of robustness of the proposed framework. This remains an important direction for future work that aims to evaluate the feasibility of real-world IoT deployment. We have explicitly stated this as a limitation of our work and clarified the reason behind choosing a discrete-event simulator. In addition, we have revised several parts of the results analysis section to provide a more cautious discussion of real world applicability, taking this limitation into account.

Author action: Clarified the choice of the discrete-event simulator in Simulation Settings section (Page 25) and added real-world deployment testing as a future direction, along with a discussion of the limitations of the current simulator in the Conclusion Section (Page 39).

Reviewer#1, Concern #2: *Statistical rigor.* Although experiments are repeated multiple times and results are averaged, the analysis remains largely descriptive. The manuscript does not report confidence intervals, variance, or statistical significance for the performance comparisons. In addition, there is no sensitivity or ablation analysis to isolate the contribution of individual design choices (e.g., periodic group selection versus the ML-based ranking itself). This makes it difficult to draw strong causal conclusions about which aspects of PoPI are responsible for the observed improvements.

Author response: Thank you for your thoughtful observations.

As noted by the reviewer, each experiment was conducted over 100 independent runs, and we reported the mean values for all evaluated metrics. We agree that including measures of variability would further strengthen the validity of the results. Therefore, to demonstrate consistency, we have included a new figure (Fig 5) for the first metric, block latency, showing the distribution of results across 100 runs at 1000 nodes. The figure shows that the relative performance ordering remains generally stable across runs. Additionally, we added a new table that reports the variance, standard deviation, and 95% confidence intervals for all consensus mechanisms. The dispersion of PoPI across 100 runs remains minimal, with a variance of 0.000252, a standard deviation of 0.015865, and a 95% confidence interval of (0.24685, 0.25315). Overall, the results confirm both the consistency of the observed improvements and the stability of the simulation environment. Since all run-level data were already available, no additional experiments were required.

To better understand the contribution of individual components of PoPI, we conduct an ablation study by isolating its three key components: ML based ranking (MR), dynamic features (DF), and the periodic grouping (PG) mechanism. The results are presented in a new table (Table 2), and the relevant discussion is added in a new subsection called Ablation Study in the Result Analysis section. A short summary of the discussion is provided here.

The results show that removing any component reduces throughput from the full system value of 610 tps. PG has the largest impact, with throughput dropping to 234 tps when removed while DF is still used, due to communication overhead from frequent DF sharing, highlighting the importance of combining DF with PG. Removing both PG and DF but retaining MR increases throughput to 468 tps because no communication is required. Excluding only DF or only MR reduces throughput to 548 tps and 567 tps from 610 tps, respectively. These findings indicate that while PG drives the largest immediate performance gain, MR and DF provide complementary benefits in several aspects, and that PoPI's improvement arises from the combined effect of all three components.

Author action: Added a new figure (Fig 5) and table (Table 1), along with relevant discussion (Page 29 and 30), to provide a statistical summary across different experimental runs. Performed an ablation study in a new table (Table 2) and a new section (Page 34 and 35) to isolate key components and clarify their individual contributions to PoPI.

Reviewer#1, Concern #3: *Machine learning component.* While PoPI is positioned as an ML-based consensus mechanism, the evaluation relies solely on a linear regression model with fixed features. There is no comparison across different model classes, no feature importance analysis, and no discussion of issues such as label noise or delayed failure outcomes. Consequently, it remains unclear how much of the performance gain is due to machine learning, as opposed to the group-based scheduling and randomization strategy.

Author response: Thank you for highlighting this point. We adopt linear regression due to its simplicity, interpretability, and low computational overhead. In practice, the choice of the underlying model should depend on application-specific requirements, as different use cases impose varying reliability and efficiency constraints. However, we acknowledge that since PoPI is designed for deployment in resource-constrained IoT environments, it is important to evaluate the resource consumption characteristics of different models to understand their feasibility in such settings. To provide a comparative overview, we summarize the model size, CPU time and inference time of Linear Regression (LR) alongside four other regression models: Ridge Regression (RR), Gradient Boosting Regressor (GBR), Random Forest Regressor (RFR), and Support Vector Regressor (SVR) in a new table (Table 3). To understand the scalability of each model, we compare these metrics for 400, 700, and 1000 nodes.

The table shows that lightweight models such as LR and RR maintain nearly constant model size, CPU time, and inference time as the node count increases, demonstrating stable and scalable behavior. Gradient Boosting Regressor (GBR) exhibits similar computational efficiency with a slightly larger memory footprint. In contrast, more complex models such as RFR and SVR require significantly higher computational resources, and their resource usage increases approximately linearly with the number of nodes. These models are generally used when complex feature interactions exist that cannot be captured by simpler models [1]. Therefore, we recommend lightweight and scalable models such as LR, RR, or GBR, provided that their predictive performance satisfies the application requirements.

Regarding whether the performance gain is attributable to the machine learning component, as discussed in response to Concern 2, the ablation study shows that excluding the machine learning module reduces throughput from 610 to 567 tps (Table 2). PoPI achieves a block latency of 250 ms (0.25 s) at 1000 nodes, implying that the 0.4 ms inference time of LR at 1000 nodes (as shown in Table 3) contributes only about 0.16% to the overall latency. This demonstrates that while the machine learning component minimally increases block latency, it contributes meaningfully to the observed performance improvement.

Author action: Added a new table (Table 3) along with a dedicated section (Page 36-38) to discuss the resource consumption of different models and clarify the rationale behind the model choice. Also clarified the performance gains attributable to machine learning by conducting a new ablation study (Page 34-35).

Reviewer#1, Concern #4: *Security considerations.* The security discussion is reasonable at a high level but rests on optimistic assumptions about supervisor honesty and the opacity of the ML model. Potential adversarial behaviors, such as supervisor collusion, strategic misreporting of device features, or partial inference of the model, are not explored in depth. A clearer threat model and discussion of these scenarios would strengthen the contribution.

Author response: Thank you for this comment.

We agree that the security discussion of PoPI benefits from a more detailed analysis of potential adversarial behaviors. To address this concern, we have restructured the security analysis section into multiple dedicated subsections, each explicitly discussing different threat scenarios and how PoPI mitigates them. Additionally, we have added new discussions that address issues such as supervisor collusion, strategic misreporting of device features, and the limited opacity of the ML model, together with the corresponding mitigation strategies of PoPI.

Author action: Reorganized the security analysis section for better clarity and added new discussions addressing various potential security concerns (Page 22-24).

Reviewer#1, Concern #5: *Overall assessment.* In summary, the manuscript presents a technically plausible and well-motivated approach, but the current experimental and statistical evidence is not sufficient to fully support the breadth of the conclusions. Strengthening the statistical analysis, clarifying the role of the ML component, and better justifying or validating the simulation assumptions would substantially improve the paper.

Author response: We sincerely thank the reviewers for their time and effort in providing valuable suggestions to improve the quality of the paper.

Author action: We have rigorously gone through the manuscript and tried to improve it.

Reviewer#2, Concern #1: The manuscript proposes a new consensus mechanism called PoPI, which aims to solve the problem of inapplicability of existing consensus mechanisms in the integration of blockchain and Internet of Things under resource-constrained devices and dynamic network conditions. The research questions are of practical significance, however the manuscript still leaves several areas for improvement.

1. It is recommended to clarify more clearly the difference between PoPI and existing similar work. It needs to be clearly stated how PoPI addresses the limitations of mechanisms such as PoEM in dynamic environments.

Author response: Thank you for this suggestion. We acknowledge that the current manuscript does not clearly state how PoPI addresses the limitations of existing mechanisms in dynamic environments.

In the *Analysis Summary* section, we elaborate on the difference of PoPI and how it improves over these mechanisms, including PoEM and PoET. In dynamic environments specifically, the problem with PoEM is that it does not introduce any particular method to assess the latest state of IoT devices, which can change very frequently. PoEM relies on fixed hardware data monitored at the start of the device joining process. However, simply sharing these data every time is not efficient, as it introduces significant communication overhead and can degrade performance.

To strengthen this claim, we perform a new ablation study where we isolate its three key components: ML-based ranking, dynamic features, and the periodic grouping mechanism, and present the results in a new table (Table 2) with relevant discussion in a subsection called Ablation Study in the Result Analysis section. The results show that if we remove dynamic features and periodic grouping from PoPI, the throughput becomes equal to 468 tps. This value is very close to PoEM at 465 tps, as the resulting configuration becomes nearly identical to PoEM. When we use dynamic features but without the periodic nature of PoPI, the throughput is reduced massively to 234 tps, lower than 465 tps of PoEM. This shows that adding just dynamic feature sharing to PoEM is not enough. Rather, it should be complemented by the periodic nature of PoPI. These observations demonstrate that by periodically sharing dynamic features through supervisors, PoPI effectively addresses the limitations of mechanisms such as PoEM in dynamic environments.

Author action: Added a new section for *Ablation Study* (Page 34-35) to highlight how different components of PoPI address the limitations of existing consensus mechanisms such as PoEM and elaborated the discussion in the *Analysis Summary* (Page 38-39) section to clarify the differences between PoPI and existing related work.

Reviewer#2, Concern #2: 2. Discussion of related work in the past 1-2 years should be added to the literature review, such as: (1) Potential of large language models in blockchain-based supply chain finance [J]. *Enterprise Information Systems*, 2025: 2541199 and (2) Decentralized finance (DeFi): a paradigm shift in the Fintech [J]. *Enterprise Information Systems*, 2024, 18(9): 2397630.

Author response: We thank the reviewer for this valuable comment.

We acknowledge that we unintentionally overlooked several recent contributions in the broader blockchain domain. Additionally, we recognize that the manuscript would benefit from a dedicated discussion of blockchain applications beyond cryptocurrency. Our current related work focuses primarily on machine learning within different blockchain applications. Since our proposed consensus mechanism is designed for non-cryptocurrency applications, such as healthcare, environmental monitoring, and agriculture, it is important to position the work within this broader context. To address this, we have added a dedicated subsection titled *Blockchain Beyond Cryptocurrency* in the *Related Work* section.

The first suggested paper has been incorporated into the existing Machine Learning in Blockchain subsection. The second suggested paper has been included in the newly created subsection, along with several other relevant and recent contributions in non-cryptocurrency blockchain applications.

Author action: Several new references and a new subsection, have been added in the Related Work (Page 4 and 5) Section, alongside the suggested ones, to strengthen the literature review.

Reviewer#2, Concern #3: 3. It is recommended to clearly state the specific supervised learning model used and explain the reasons for selecting this model.

Author response: Thank you for highlighting this point. We have mentioned that the use linear regression (LR) models in subsection Compared Consensus Mechanism under Experimental Design Section. We adopt LR due to its simplicity, interpretability, and low computational overhead. In practice, the choice of the underlying model should depend on application-specific requirements, as different use cases impose varying reliability and efficiency constraints.

However, we acknowledge that since PoPI is designed for deployment in resource-constrained IoT environments, it is important to evaluate the resource consumption characteristics of different models to understand their feasibility in such settings. We summarize the resource consumption characteristics of LR along with four other models: Ridge Regression (RR), Gradient Boosting Regressor (GBR), Random Forest Regressor (RFR), and Support Vector Regressor (SVR) in a new table (Table 3). The table shows that LR maintain nearly constant model size, CPU time, and inference time as the node count increases, demonstrating stable and scalable behavior. In contrast, more complex models such as RFR and SVR require significantly higher computational resources, and their resource usage increases approximately linearly with the number of nodes. These models are generally when complex feature interactions exist that cannot be captured by simpler models [1]. Therefore, we recommend lightweight and scalable models similar to LR, provided that their predictive performance satisfies the application requirements.

Author action: Added a new table (Table 3) and a new subsection (Page 36-38) to compare different machine learning models and clarified the rationale behind selecting the specific model.

Reviewer#2, Concern #4: 4. The result analysis mostly stays at the data description level, lacking an in-depth explanation of the underlying mechanism.

Author response: We thank the reviewer for pointing this out. We agree that certain parts of the *Result Analysis* section could benefit from a more detailed discussion of the underlying mechanisms behind the observed results. We have revised several portions of the existing subsections of *Result Analysis* to provide clearer and more in-depth explanations. We have also carefully ensured that the newly added content in this

section offers sufficient explanatory detail.

Author action: Revised several portions of the Result Analysis section (Page 28-34) to focus on explaining the observed values and highlighted the corresponding revisions accordingly.

Reviewer#2, Concern #5: 5. It is recommended to add specific tests for different IoT scenarios to verify the performance of PoPI in different environments.

Author response: Thank you for this valuable recommendation.

In the current experimental setup, we incorporate stochastic IoT behaviors to emulate diverse and dynamic environments. Specifically, random events such as temporary node failures, accelerated battery depletion, halted charging, and reduced connectivity are introduced during simulation. These events dynamically affect each node's ability to produce or validate blocks, thereby capturing the instability, heterogeneity, and volatility typical of real-world IoT deployments. By modeling these stochastic conditions, the evaluation inherently reflects different IoT operational scenarios, including unstable connectivity, energy-constrained environments, and fluctuating device availability. Therefore, PoPI's performance under varying IoT conditions is implicitly examined through these randomized environmental disruptions.

That said, we agree that scenario-specific case studies (e.g., smart city, industrial IoT, environmental monitoring) could further strengthen the evaluation. We have added this as a direction for future work to explore PoPI's performance under application-specific IoT deployments.

Author action: Clarified how diverse IoT device operating conditions are captured within the current simulation settings (Page 25) and added scenario-specific IoT evaluations as a direction for future work in the conclusion section (Page 39).

Reviewer#3, Concern #1: *Strengths*

- The periodic selection of a group of block producers effectively minimizes the computational overhead typically associated with running machine learning inferences for every single block.

- Incorporating dynamic device characteristics, such as real-time battery levels and network bandwidth, into the consensus selection process represents a highly practical approach to improving IoT network reliability.

- The introduction of fair participation features, such as Longevity Probability and Starvation Duration, ensures a balanced network involvement and mitigates the risk of centralization by preventing the repeated selection of the exact same nodes.

- The experimental setup provides a comprehensive comparison against relevant modern consensus protocols, specifically DT-DPoS, CE-PBFT, PPoR, PoET, and PoEM, across meaningful metrics like throughput, latency, and computational overhead.

Issue:

- The security analysis states that the machine learning model acts as a "black box," making selections unpredictable. However, because the system deterministically selects producers by running the exact same model available across all nodes, an adversary could potentially run the same inference to predict group selection and target supervisor nodes. The authors should address how the protocol prevents this deterministic predictability.

Author response: Thank you for this insightful observation.

Although the same model is available to all nodes, its weights are updated at the end of each round, prior to the selection of new producers, based on the success or failure of the previous block producers. Hence, the updated model parameters become available only immediately before the subsequent selection process, limiting any advance predictability. Additionally, even if the model parameters were known or did not change significantly, resulting in a similar candidate set, the input data required to perform inference are not globally accessible. The dynamic features of the nodes change frequently, and these updated feature values are transmitted exclusively to the designated supervisors for that round. As a result, external adversaries cannot reconstruct the exact inference inputs needed to deterministically predict group selection or target

supervisor nodes. We acknowledge that the current manuscript does not clearly articulate these critical security details, and we have revised the Security Analysis section accordingly to clarify this mechanism.

Author action: Added a discussion clarifying how PoPI avoids a deterministic prediction pattern in the ML model (Page 22-23).

Reviewer#3, Concern #2: The mechanism continuously trains the model using block production outcomes as labels, assigning a 1 for success and a 0 for failure. The authors need to clarify how the system handles false negatives or distinguishes between malicious failures and benign failures (e.g., sudden hardware drops), as mislabeling could degrade model accuracy over time.

Author response: We thank the reviewer for this important question.

In our design, the training signal is derived from block production outcomes, where success/failure can be considered a weak label [2]. This is because training labels are automatically derived from observable system outcomes (i.e., block production outcomes) rather than manually verified ground-truth annotations (i.e., network delay, temporary failure, or malicious behavior). However, a single failure does not significantly degrade a node's suitability score, as the model learns from aggregated historical behavior rather than isolated events. Since model updates are performed over multiple rounds with accumulated data, occasional false negatives have limited impact and are naturally smoothed out during training. If a node suffers from benign failours too often, even if it is not a malicious failures, it is better to exclude the node from selection to maintain system liveness. Therefore, benign transient failures are unlikely to be permanently penalized, whereas persistent malicious behavior is reflected through consistent negative outcomes.

Author action: Added a discussion on how labels based on block production outcomes distinguish between malicious failures and benign failures (Page 20).

References

[1] U. Grömping, "Variable importance assessment in regression: linear regression versus random forest," *The American Statistician*, vol. 63, no. 4, pp. 308–319, 2009.

[2] Q.-W. Wang, L. Yang, and Y.-F. Li, "Learning from weak-label data: A deep forest expedition," in *Proceedings of the AAAI Conference on Artificial Intelligence*, vol. 34, no. 04, 2020, pp. 6251–6258.
